# Fkh1 and Fkh2 associate with Sir2 to control *CLB2* transcription under normal and oxidative stress conditions

**DOI:** 10.3389/fphys.2013.00173

**Published:** 2013-07-12

**Authors:** Christian Linke, Edda Klipp, Hans Lehrach, Matteo Barberis, Sylvia Krobitsch

**Affiliations:** ^1^Otto Warburg Laboratory, Department of Vertebrate Genomics, Max Planck Institute for Molecular GeneticsBerlin, Germany; ^2^Department of Biology, Chemistry and Pharmacy, Free University BerlinBerlin, Germany; ^3^Theoretical Biophysics, Institute for Biology, Humboldt University BerlinBerlin, Germany; ^4^Dahlem Centre for Genome Research and Medical Systems BiologyBerlin, Germany; ^5^Synthetic Systems Biology and Nuclear Organization, Swammerdam Institute for Life Sciences, University of AmsterdamAmsterdam, Netherlands

**Keywords:** Fkh1, Fkh2, Sir2, silencing, cell cycle, stress, budding yeast

## Abstract

The Forkhead (Fkh) box family of transcription factors is evolutionary conserved from yeast to higher eukaryotes and its members are involved in many physiological processes including metabolism, DNA repair, cell cycle, stress resistance, apoptosis, and aging. In budding yeast, four Fkh transcription factors were identified, namely Fkh1, Fkh2, Fhl1, and Hcm1, which are implicated in chromatin silencing, cell cycle regulation, and stress response. These factors impinge transcriptional regulation during cell cycle progression, and histone deacetylases (HDACs) play an essential role in this process, e.g., the nuclear localization of Hcm1 depends on Sir2 activity, whereas Sin3/Rpd3 silence cell cycle specific gene transcription in G2/M phase. However, a direct involvement of Sir2 in Fkh1/Fkh2-dependent regulation of target genes is at present unknown. Here, we show that Fkh1 and Fkh2 associate with Sir2 in G1 and M phase, and that Fkh1/Fkh2-mediated activation of reporter genes is antagonized by Sir2. We further report that Sir2 overexpression strongly affects cell growth in an Fkh1/Fkh2-dependent manner. In addition, Sir2 regulates the expression of the mitotic cyclin Clb2 through Fkh1/Fkh2-mediated binding to the *CLB2* promoter in G1 and M phase. We finally demonstrate that Sir2 is also enriched at the *CLB2* promoter under stress conditions, and that the nuclear localization of Sir2 is dependent on Fkh1 and Fkh2. Taken together, our results show a functional interplay between Fkh1/Fkh2 and Sir2 suggesting a novel mechanism of cell cycle repression. Thus, in budding yeast, not only the regulation of G2/M gene expression but also the protective response against stress could be directly coordinated by Fkh1 and Fkh2.

## Introduction

Transcription factors play essential roles in modulating gene expression implicated in multiple cellular processes. Among them, Forkhead (Fkh) transcription factors are highly conserved in eukaryotes and have been intensively studied because of their involvement in diverse cellular processes, such as cell cycle regulation, apoptosis, DNA damage, cellular development and differentiation, metabolism, oxidative stress, and aging (Laoukili et al., 2007; Tuteja and Kaestner, 2007a; van der Horst and Burgering, 2007; Fu and Tindall, 2008; Hannenhalli and Kaestner, 2009; Kloet and Burgering, 2011; Storz, 2011; Zhang et al., 2011; Postnikoff et al., 2012; Sandri, 2012; van der Vos et al., 2012). In addition, many of them play important roles in cancer and other human diseases. Hitherto, the mammalian Fkh family comprises 18 subfamilies (Tuteja and Kaestner, 2007a,b), of which two, the FOX class O and M, are conserved in the budding yeast *Saccharomyces cerevisiae*. In this regard, FoxO and FoxM proteins represent the closest functional homologs of the yeast Fkh transcription factors Hcm1 and Fkh1/2, respectively, which are also implicated in cell cycle regulation, stress response, chromatin silencing, and aging (Murakami et al., 2010).

In budding yeast, Fkh1 and Fkh2 play an essential role by binding and activating a cluster of genes that encode proteins including the Clb2 cyclin (*CLB2*-cluster genes), which drives progression through mitosis after binding to the Cdk1 kinase (Breeden, 2000; Futcher, 2000). Fkh2 is the major regulator of Clb2, repressing its transcription in G1 phase and stimulating its expression in late S and G2/M phase through binding to the coactivator Ndd1 (Koranda et al., 2000; Kumar et al., 2000; Pic et al., 2000; Zhu et al., 2000; Reynolds et al., 2003). Fkh1 can functionally complement the absence of Fkh2, although it binds less efficiently to the *CLB2* promoter, but its primary role is the repression of *CLB2* transcription (Hollenhorst et al., 2000, 2001; Sherriff et al., 2007), thus competing with Fkh2 for the *CLB2* promoter occupancy. These opposite effects of Fkh2 as an activator and Fkh1 as a repressor balance Clb2 level, which is critical to drive cell division. Compared to wild type, *fkh2*Δ cells exhibit a delay in cell cycle progression and a reduced *CLB2* mRNA level, whereas *fkh1*Δ cells show an anticipated cell cycle progression and enhanced *CLB2* mRNA level (Hollenhorst et al., 2000; Casey et al., 2008). Although the role of Fkh1 in transcriptional processes is not well understood, recent evidence suggest that this transcription factor is involved in the silencing of the mating-type locus *HMR* by stabilizing the binding of the silent information regulator (Sir) proteins or sirtuins (Hollenhorst et al., 2000), a protein family known to regulate silencing, chromatin organization, DNA repair, cell cycle regulation, and aging (Guarente, 1999). Moreover, Hcm1, required for the activation of Fkh1 and Fkh2 (Pramila et al., 2006), has been shown to interact with Sir2, which activity regulates its nuclear localization and therefore Hcm1-mediated gene expression (Rodriguez-Colman et al., 2010). In addition, Fkh2 represses *CLB2* transcription during early phases of the cell cycle (Koranda et al., 2000; Zhu et al., 2000) via interaction with the Sin3/Rpd3 histone deacetylase (HDAC) complex (Hollenhorst et al., 2000; Ho et al., 2002). This silencing complex is assembled at the *CLB2* promoter via Fkh2 to promote a repressive nucleosomal structure at the M/G1 transition (Veis et al., 2007; Voth et al., 2007). Altogether, these findings indicate that Fkh transcription factors regulate both positively and negatively the Clb2 level critical for cell division, and suggest that Fkh-dependent recruitment of HDAC could be an essential mechanism to control chromatin silencing.

Interestingly, the induction of oxidative stress in yeast via hydrogen peroxide (H_2_O_2_) or menadione (MD) results in cell cycle arrest (Flattery-O'Brien and Dawes, 1998), and the transcriptional response is mediated by Fkh transcription factors (Shapira et al., 2004). Deletion or overexpression of both *FKH1* and *FKH2* impact stress resistance as well as chronological and replicative lifespan of yeast cells (Postnikoff et al., 2012). Additionally, Hcm1 deficiency causes reduced viability of yeast cells upon H_2_O_2_ or MD treatment, and its overexpression leads to increased stress resistance (Rodriguez-Colman et al., 2010). Under normal conditions, Hcm1 shifts from the cytoplasm to the nucleus during G1/S phase, but nuclear translocation is enhanced under oxidative stress conditions (Rodriguez-Colman et al., 2010).

In this study we explored whether Fkh1/Fkh2 and Sir2 are involved in silencing cell cycle genes and whether Fkh transcription factors play a protective role against oxidative stress mediated by Sir2, as shown for Hcm1 (Rodriguez-Colman et al., 2010). We were able to demonstrate a functional interplay between Fkh1/Fkh2 and Sir2 in G1 and M phase. Moreover, we found that Sir2 antagonizes the Fkh1/Fkh2-mediated regulation of the mitotic cyclin Clb2 through binding to the *CLB2* promoter via Fkh1 and Fkh2. Therefore, Fkh1/Fkh2-mediated chromatin silencing might provide an additional level of regulation of the cell division cycle, which is also required for an increased stress resistance, as previously suggested for Fkhs (Shapira et al., 2004; Rodriguez-Colman et al., 2010; Postnikoff et al., 2012).

## Materials and methods

### Yeast strains and growth conditions

Yeast strains BY4741 (*MATa his3Δ1 leu2Δ0 met15Δ0 ura3Δ0*) and L40ccua (*MATa his3-200 trp1-901 leu2-3,112 LYS2::(lexAop)4-HIS3 ura3::(lexAop)8-lacZ ADE2::(lexAop)8-URA3 gal80 canR cyh2R*) were used to generate the respective strains in this study (Table 1). Generally, a one-step PCR-mediated gene targeting procedure was carried out for genetic manipulations (Longtine et al., 1998), and oligonucleotides used in this study are listed in Table 2. To generate the respective gene deletion strains, the plasmid pUG6 (accession number P30114, Euroscarf) served as template to amplify a gene-specific loxP-flanked G418 cassette. For the deletion of FKH1 or FKH2, the oligonucleotide pair Fwd-fkh1Δ and Rev-fkh1Δ, or Fwd-fkh2Δ and Rev-fkh2Δ was used. Disruption of SIR2 was achieved using the oligonucleotide pair Fwd-sir2Δ and Rev-sir2Δ. Then, the amplified DNA cassettes were used for transformation (Longtine et al., 1998). After selection of transformants and verification of the correct chromosomal integration of the loxP-flanked cassette, a respective yeast clone was transformed with plasmid pSH47 (accession number P30119, Euroscarf) to express Cre-recombinase for excision of the integrated gene-specific loxP-flanked cassette. Subsequently, transformants were incubated on selective medium containing 1 mg/ml 5-fluoroorotic acid (Zymo Research), and grown yeast clones were analyzed for uracil auxotrophy. To generate double gene deletions, a second integration cassette was amplified using plasmid pUG6 as template and used to transform the corresponding deletion strains. To express Myc-tagged proteins in yeast, plasmid pYM18 containing a 9-MYC sequence (accession number P30304, Euroscarf) served as template for the amplification of the respective gene-specific integration cassettes. Chromosomally MYC-tagged FKH1 or FKH2 was accomplished by using oligonucleotides Fwd-Fkh1-Myc and Rev-Fkh1-Myc, or Fwd-Fkh2-Myc and Rev-Fkh2-Myc. MYC-tagged HCM1 was achieved using the oligonucleotides Fwd-Hcm1-Myc and Rev-Hcm1-Myc.

**Table 1:** **Yeast strains used in this study**.

**Strain**	**Genotype**	**Source**
L40ccua	*MATa his3_200 trp1-901 leu2-3112*	Goehler et al., 2004, Ralser et al., 2005
	*LYS2*::*(lexAop)4-HIS3 ura3*::*(lexAop)8-lacZ*	
	*ADE2*::*(lexAop)8-URA3 gal80 canR cyh2R*	
L40ccua *sir2*Δ	*MATa sir2*::*kanMX6*	This study
BY4741	*MATa his3Δ1 leu2Δ0 met15Δ0 ura3Δ0*	Euroscarf
Fkh1-Myc	*MATa FKH1-MYC9*::*kanMX6*	This study
Fkh2-Myc	*MATa FKH2-MYC9*::*natNT2*	This study
Hcm1-Myc	*MATa HCM1-MYC9*::*kanMX6*	This study
Sir2-Myc	*MATa SIR2-MYC9*::*kanMX6*	This study
*fkh1*Δ Sir2-Myc	*MATa fkh1*:: *SIR2-MYC9*::*kanMX6*	This study
*fkh2*Δ Sir2-Myc	*MATa fkh2*:: *SIR2-MYC9*::*kanMX6*	This study
*fkh1*Δ	*MATa fkh1*::	This study
*fkh2*Δ	*MATa fkh2*::	This study
*fkh1*Δ *fkh2*Δ	*MATa fkh1*:: *fkh2*::	This study
*sir2*Δ	*MATa sir2*::*kanMX6*	This study
*fkh1*Δ *sir2*Δ	*MATa fkh1*:: *sir2*::*kanMX6*	This study
*fkh2*Δ *sir2*Δ	*MATa fkh2*:: *sir2*::*kanMX6*	This study
Sir2-EGFP	*MATa SIR2-EGFP*::*kanMX6*	This study
Sir2-EGFP *fkh1*Δ	*MATa fkh1*:: *SIR2-EGFP*::*kanMX6*	This study
Sir2-EGFP *fkh2*Δ	*MATa fkh2*:: *SIR2-EGFP*::*kanMX6*	This study
Sir2-VC/VN-Fkh1	*MATa SIR2-VC*::*kanMX6* p426GPDpr-*VN-FKH1*	This study
Sir2-VC/VN-Fkh2	*MATa SIR2-VC*::*kanMX6* p426GPDpr-*VN-FKH2*	This study
Sir2-VC/VN-Hcm1	*MATa SIR2-VC*::*kanMX6* p426GPDpr-*VN-HCM1*	This study
Ndd1-VC/VN-Fkh2	*MATa NDD1-VC*::*kanMX6* p426GPDpr-*VN-FKH2*	This study

**Table 2:** **Oligonucleotides used in this study**.

**Primer**	**Sequence**
Fwd-*fkh1*Δ	5′-TGTGCGTTCAATTAGCAAAGAAAGGCTTGGAGAGACACAGGTACGCTGCAGGTCGACAAC-3′
Rev-*fkh1*Δ	5′-TATTGTTTAATAATACATATGGGTTCGACGACGCTGAATTCTAGTGGATCTGATATCACC-3′
Fwd-*fkh2*Δ	5′-GTGCTCCCTCCGTTTCCTTTATTGAAACTTTATCAATGCGGTACGCTGCAGGTCGACAAC-3′
Rev-*fkh2*Δ	5′-TTCATTTCTTTAGTCTTAGTGATTCACCTTGTTTCTTGTCCTAGTGGATCTGATATCACC-3′
Fwd-*sir2*Δ	5′-CATTCAAACCATTTTTCCCTCATCGGCACATTAAAGCTGGGTACGCTGCAGGTCGACAAC-3′
Rev-*sir2*Δ	5′-TATTAATTTGGCACTTTTAAATTATTAAATTGCCTTCTACCTAGTGGATCTGATATCACC-3′
Fwd-Fkh1-Myc	5′-CGTAACAACAAACGCAAACGTGAACAATTCCTCTCTGAGTGCTAGTGGTGAACAAAAG-3′
Rev-Fkh1-Myc	5′-TATTGTTTAATAATACATATGGGTTCGACGACGCTGAATTTAGTGGATCTGATATCATCG-3′
Fwd-Fkh2-Myc	5′-ACTAGATACGGATGGTGCAAAGATCAGTATTATCAACAACGCTAGTGGTGAACAAAAG-3′
Rev-Fkh2-Myc	5′-TTCATTTCTTTAGTCTTAGTGATTCACCTTGTTTCTTGTCTAGTGGATCTGATATCATCG-3′
Fwd-Hcm1-Myc	5′-TCATAATCACCCTTCCAACGATAGCGGTAATGAAAAGAATGCTAGTGGTGAACAAAAG-3′
Rev-Hcm1-Myc	5′-CAACCGTTTGCGATGAATCCATCAGATTAAGAATAATTAGTAGTGGATCTGATATCATCG-3′
Fwd-Sir2-GFP	5′-CGTGTATGTCGTTACATCAGATGAACATCCCAAAACCCTCGGAGCAGGTGCTGGTGCTGG-3′
Rev-Sir2-GFP	5′-TATTAATTTGGCACTTTTAAATTATTAAATTGCCTTCTACCTAGTGGATCTGATATCATCG-3′
Fwd-Sir2-VC	5′-CGTGTATGTCGTTACATCAGATGAACATCCCAAAACCCTCGGTCGACGGATCCCCGGGTT-3′
Rev-Sir2-VC	5′-TATTAATTTGGCACTTTTAAATTATTAAATTGCCTTCTACTCGATGAATTCGAGCTCGTT-3′
Fwd-Ndd1-VC	5′CTGTAATTCTAAATCTAATGGAAATTTATTCAATTCACAGGGTCGACGGATCCCCGGGTT-3′
Rev-Ndd1-VC	5′-TCGATTAAAAAAAAAAGGTGAGATGCAAGTTTGGTTAATATCGATGAATTCGAGCTCGTT-3′
Fwd-F1s	5′-GTCAGTCGACTATGTCTGTTACCAGTAG-3′
Rev-F1n	5′-AATGCGGCCGCTGAATTTCAACTCAG-3′
Fwd-F2s	5′-TGAAGTCGACAATGTCCAGCAGCAAT-3′
Rev-F2n	5′-ATTGCGGCCGCTTAGTTGTTGATAATAC-3′
Fwd-H1s	5′-TTGAGTCGACAATGATGAATGAAG-3′
Rev-H1n	5′-TTGCGGCCGCTCAATTCTTTTCATTACC-3′
Fwd-Vb	5′-TAGGATCCATGGTGAGCAAGGGCG-3′
Rev-Ve	5′-TCGAATTCCTCGATGTTGTGGCGGAT-3′
Fwd-F1e	5′-AGAATTCTCGACTATGTCTGTTACC-3′
Rev-F1x	5′-CTCTCGAGTCAACTCAGAGAGGAATTG-3′
Fwd-F2e	5′-AGAATTCTCGACAATGTCCAGCAGC-3′
Rev-F2x	5′-GTCTCGAGTTAGTTGTTGATAATACTG-3′
Fwd-H1s	5′-TTGAGTCGACAATGATGAATGAAG-3′
Rev-H1n	5′-TTGCGGCCGCTCAATTCTTTTCATTACC-3′
*TSA1* ORF	5′-ATGGTCGCTCAAGTTCAAAAG-3′
	5′-CGTACTTACCCTTGTATTTGTCCAA-3′
*ACT1* ORF	5′-ATGTGTAAAGCCGGTTTTGC-3′
	5′-TGACCCATACCGACCATGATA-3′
*CLB2* ORF	5′-GGAATGTACAAGGTTGG-3′
	5′-CAAATTGCTGACTACTTGG-3′
*CLB2* promoter	5′-GTGCAAGTTCAAGGCAC-3′
	5′-CATGCTATGAGATGCTAG-3′

For tagging *SIR2* with the *GFP* sequence, plasmid pYM27-EGFP (accession number P30239, Euroscarf) and oligonucleotides Fwd-Sir2-GFP and Rev-Sir2-GFP were used. Plasmid pFA6a-Venus-C (accession number EF210810, Sung and Huh, 2007) and oligonucleotides Fwd-Sir2-VC and Rev-Sir2-VC were used to generate the Sir2-Venus-C fusion cassette. The Ndd1-Venus-C fusion construct was amplified from plasmid pFA6a-Venus-C using oligonucleotides Fwd-Ndd1-VC and Rev-Ndd1-VC. Genetic manipulations of all strains generated in this study were validated by PCR analysis.

Yeast strains were grown in yeast extract-peptone-dextrose (YPD) or synthetic complete (SC) media (0.67 g/l Yeast Nitrogen Base-ADE-HIS-LEU-TRP-URA, Difco Laboratories; 0.59 g/l Complete Supplement Mixture-ADE-HIS-LEU-TRP-URA, MP Biomedicals, LLC) containing 2% glucose as carbon source with respective antibiotic and auxothrophic additives at 30°C. For life-span experiments, the respective yeast strains were incubated in YPD overnight (OD_600_ ~1.6) and subsequently collected by centrifugation. Then, yeast cells were washed twice with water and further incubated in water for 3 weeks with cells washed every 48 h with water to remove metabolic byproducts or nutrients released from dead cells. Samples were taken each week, and cells were spotted in serial dilutions on SC medium containing all auxotrophic supplements and 2% glucose as carbon source.

### Plasmids

Plasmids pBTM117c-Fkh1, pBTM117c-Fkh2, and pBTM117c-Hcm1 encoding the fusion proteins LexA-Fkh1, LexA-Fkh2, and LexA-Hcm1, respectively, were generated as described in the following, and oligonucleotides used in this study are listed in Table 2. The open reading frame of *FKH1* was amplified using genomic DNA isolated from BY4741 as template and primers Fwd-F1s and Rev-F1n. For the amplification of the open reading frames of *FKH2* and *HCM1*, primer pairs Fwd-F2s and Rev-F2n or Fwd-H1s and Rev-H1n were used. The amplified DNA fragments were purified and subcloned into the cloning vector pJET1.2/blunt (CloneJET PCR Cloning Kit, Fermentas). Subsequently, the sequence of the obtained constructs was validated by sequencing, verified plasmid DNA was then treated with the restriction endonucleases *Sal*I and *Not*I, purified and subcloned into the *Sal*I/*Not*I sites of pBTM117c.

For the construction of the plasmid p426GPD-VN encoding the N-terminal region of the Venus protein, a PCR was performed using plasmid pFA6a-Venus-N (accession number EF210809, Sung and Huh, 2007) as DNA template and primer pair Fwd-Vb and Rev-Ve. Subsequently, the resultant DNA fragment was subcloned into the vector pJET1.2/blunt to generate plasmid pJET1.2-VN. After sequence validation, plasmid pJET1.2/VN was treated with *Bam*HI and *Eco*RI, and the resultant DNA fragment was ligated into the *Bam*HI/*Eco*RI sites of vector p426GPD (Mumberg et al., 1995).

Plasmids p426GPD-VN-Fkh1, p426GPD-VN-Fkh2, and p426GPD-VN-Hcm1 were generated to express N-terminal Venus-N-tagged Fkh1, Fkh2, and Hcm1. For this purpose, the open reading frame of *FKH1, FKH2*, and *HCM1* was amplified using genomic DNA isolated from BY4741 as template and primer pairs Fwd-F1e and Rev-F1x or Fwd-F2e and Rev-F2x or Fwd-H1s and Rev-H1n. After PCR, resultant DNA fragments were subcloned into vector pJET1.2/blunt. Subsequently, sequences were validated, and the respective plasmid DNA was treated with *Eco*RI and *Xho*I (for *FKH1* and *FKH2*) or with *Sal*I and *Not*I (for *HCM1*). After purification, the DNA fragments were subcloned into the *Eco*RI/*Xho*I or *Sal*I/*Not*I sites of plasmid p426GPD-VN.

Plasmids p423GAL-Sir2, encoding the open reading frame of *SIR2* under control of the GAL promoter, and pGEX6p2-Sir2, encoding the open reading frame of *SIR2* fused to *GST*, were generated by treating plasmid pACT4-1b-Sir2 with *Sal*I and *Not*I. After isolation, the respective DNA fragments were subcloned into the *Sal*I/*Not*I sites of vectors p423GAL (Mumberg et al., 1995) or pGEX6p2 (Phamarcia Biotech). Plasmid p423GAL-Clb2 was generated by treating plasmid pBTM117c-Clb2 with *Sal*I and *Not*I. Afterwards, the DNA fragment was purified and subcloned into the *Sal*I / *Not*I sites of vector p423GAL.

### GST pull-down

Pull-down assays were performed as previously described (Barberis et al., 2012). Briefly, *E. coli* strain XL1blue (*endA1 gyrA96(nal^R^) thi-1 recA1 relA1 lac glnV44 F′[::Tn10 proAB^+^ lacI^q^ Δ(lacZ)M15] hsdR17(r_K−_ m_K+_)*; Stratagene) was transformed with plasmid pGEX6p2-Sir2 and incubated in LB media. At OD_600_ of ~0.5–0.7, expression of proteins was induced by adding 1 mM isopropylbeta-D-thiogalactopyranoside (IPTG, Fermentas), and cultures were incubated for additional 3 h at 37°C. Subsequently, cells were harvested and lysed in GST-binding buffer (20 mM TrisHCl pH 7.9, 125 mM NaCl, 5 mM MgCl_2_, 0.5 mM DTT, 10 mg/ml Lysozym; Sigma-Aldrich). Then, cell lysates were sonicated 10 times for 10 s (Branson Sonifier W250), and 10% Glycerol and 0.1% NP-40 were added. After centrifugation (25 min, 20,000 rcf, 4°C), Glutathione Sepharose 4B beads (GE Healthcare) were added to the supernatants containing the expressed GST-tagged proteins and incubated for 8 h at 4°C. Then, beads were washed with GST-binding buffer, added to 1 ml yeast protein lysates (5 μg/μl total protein), which were prepared from yeast cells expressing Myc-tagged Fkh1, Fkh2, or Hcm1, and incubated overnight at 4°C. Briefly, 200 ml of yeast cultures (OD_600_ ~0.7) were harvested by centrifugation, cell pellets were washed with phosphate-buffered saline solution (PBS; 137 mM NaCl, 2.7 mM KCl, 10 mM Na_2_HPO_4_, 2 mM KH_2_PO_4_, pH 7.4), frozen in liquid nitrogen and lysed with glass beads (acid-washed, 425–600 μm in diameter, Sigma-Aldrich) by vigorous shaking. Finally, pull-down samples were washed twice with ice-cold GST-binding buffer and bound proteins were eluted with SDS sample buffer.

### Western blot

For GST pull-down assays, protein samples were loaded and separated using 10% Sodium Dodecyl Sulphate (SDS) gels. Subsequently, proteins were transferred onto a nitrocellulose Protran membrane (PerkinElmer), and membranes were treated with rabbit α-Myc antibody (1:10,000, Sigma- Aldrich) and with the corresponding peroxidase (POD)-coupled secondary antibody (1:5000, α-rabbit IgG POD conjugate, Sigma-Aldrich). For the analysis of yeast protein extracts, 20 ml cultures of wild type or deletion strains were centrifuged and washed with 1 × PBS. Cells were then lysed with glass beads by vigorous shaking and cleared by centrifugation (1 min, 10,000 rcf, 4°C). Then, protein lysates were loaded onto a 10% SDS gel, and proteins were separated by SDS-PAGE and transferred onto a nitrocellulose Protran membrane (PerkinElmer). Subsequently, membranes were treated with rabbit α-Clb2 antibody (1:1000, Santa Cruz Biotechnology) and the corresponding POD-coupled secondary antibody (1:5000, α-rabbit IgG POD conjugate, Sigma-Aldrich). Membranes were treated with Western Lighting luminol reagent (PerkinElmer) and exposed to a high performance chemiluminescence film (GE Healthcare) to visualize proteins. In addition, gels were incubated in staining solution (40% Methanol, 7% Acetic acid, 0.1% Coomassie Brilliant Blue R250) to verify an equal loading of samples. De-staining of gels was performed in 40% Methanol and 10% Acetic acid.

### Bimolecular fluorescence complementation (BiFC) and fluorescence microscopy

Haploid yeast cells expressing the C-terminal region of the Venus protein fused to the C-terminal region of Sir2 (Sir2-VC) or Ndd1 (Ndd1-VC) were transformed either with plasmids p426GPD-VN-Fkh1, p426GPD-VN-Fkh2, or p426GPD-VN-Hcm1 encoding fusion proteins between the N-terminal region of Venus and the C-terminal region of the selected transcription factors. Subsequently, yeast clones were isolated and cultured in liquid SC media under different experimental conditions. Imaging of haploid cells expressing GFP-tagged Sir2 grown in YPD medium was performed at stationary phase (OD_600_ ~1.6). Staining of the nucleus was performed by adding 2.5 μg/ml 4′,6-diamidino-2-phenylindole (DAPI, Sigma-Aldrich) to the media. After 20 min, cells were harvested by centrifugation, washed once with 1 × PBS and monitored for a Venus-dependent BiFC signal using a Zeiss AxioImager Z1 microscope (Carl Zeiss AG, Germany) with a Plan-NeoFluar 60 ×/1.3 NA oil immersion objective. Fluorescence images were taken using a standard fluorescein isothiocyanate filter set (excitation band pass filter, 450–490 nm; beam splitter, 510 nm; emission band pass filter, 515–565 nm), and recorded on a Zeiss Axiocam Mrm (Carl Zeiss AG) with 2 × 2 binning.

### Reporter gene assay

L40ccua and L40ccua/*sir2*Δ cells were transformed with the respective pBTM117c and pACT4-1b plasmids as indicated. Transformants were selected on SC SDII medium lacking tryptophan (TRP^−^) and leucine (LEU^−^). Subsequently, overnight cultures were spotted in 1:5 serial dilutions on SDII and SDIV (TRP^−^, LEU^−^, HIS^−^ and URA^−^) media. Plates were incubated for 5 days at 30°C, and cell growth was monitored.

For measuring β-galactosidase activity, protein extracts were prepared from 50 ml of yeast cultures (OD_600_ ~0.7). Then, cells were harvested, washed with 1 × PBS and lysed with glass beads by vigorous shaking in 1 × PBS supplemented with protease inhibitor cocktail (Roche Diagnostics GmbH). After centrifugation (1 min, 10,000 rcf, 4°C), equal amounts of protein lysates were added to 500 μl Z-buffer (10.7 g/l Na_2_HPO_4_·2 H_2_O, 5.5 g/l NaH_2_PO_4_·1 H_2_O, 0.75 g/l KCL, 0.246 g/l MgSO_4_·7 H_2_O, pH 7) supplemented with 0.02% X-Gal (Sigma-Aldrich) and 20 mM DTT (Sigma-Aldrich). Samples were incubated for 4 h at 37°C and the colorimetric assay was performed at 420 nm (Spectrophotometer 6700 Vis., Jenway).

### Cell synchronization

For synchronization experiments, overnight cultures of yeast strains were diluted to an OD_600_ ~0.1–0.2 and incubated to reach an OD_600_ ~0.6. To induce cell cycle arrest in G1 phase, cells were treated with α-factor (15 μg/ml, Universitat Pompeu Fabra, Barcelona) and further incubated for 2.5 h at 30°C. Arrest of cells in S phase or metaphase was achieved by adding 75 mM hydroxyurea (Sigma-Aldrich) or 5 μg/ml nocodazole (AppliChem), respectively. Subsequently, cells were incubated for additional 2 h at 30°C. In time course experiments, α-factor was added to the cultures and arrested cells were harvested after 2 h. Cell pellets were washed twice with medium. After addition of fresh medium, samples were taken every 10 min and analyzed by fluorescence microscopy and flow cytometry (FACS).

To analyze yeast growth under oxidative stress conditions, 2 mM H_2_O_2_ (Sigma-Aldrich) or 40 μM MD (Sigma-Aldrich) was added to solid medium or liquid medium, in which cells were incubated for additional 90 min at 30°C.

### Flow cytometry

For FACS analysis, cells were fixed in 70% ethanol and treated overnight with RNAse A (0.25 mg/ml final concentration, Sigma-Aldrich) and Proteinase K (0.5 mg/ml final concentration, Sigma-Aldrich) in 50 mM sodium citrate. DNA was stained with propidium iodide, and a total of 10,000 cells were analyzed in a flow cytometer (FACSCalibur, Becton Dickinson Immunocytometry Systems, USA).

### Chromatin immunoprecipitation

Chromatin immunoprecipitation (ChIP) assays were performed as follows. Briefly, yeast cells expressing Myc-tagged Sir2 were cross-linked with 1% formaldehyde (16% solution in methanol-free water, Ultra Pure EM Grade, Polysciences Inc.). Then, cells were harvested by centrifugation and cell pellets were resuspended in pre-cooled lysis buffer (50 mM HEPES/KOH, pH 7.5, 500 mM NaCl, 1 mM EDTA, 1% Triton X-100, 0.1% DOC, 0.1% SDS, complete protease inhibitor cocktail, Roche Diagnostics GmbH). Glass beads (acid-washed, 425–600 μm in diameter, Sigma-Aldrich) were added to the samples, which were then vortexed 3 times for 90 s. Subsequently, the soluble protein-DNA fraction was sonicated 3 times for 10 s (Branson Sonifier W250). For the immunoprecipitation, 10 μg goat α-Myc antibody (1:1000, Abcam) were added to cell lysates, which were then incubated on a rotation wheel for additional 2 h at 4°C. To immobilize the immune complex, 50 μl of pre-cooled Protein A/G agarose mix in 1 × PBS (50% mix of Protein A/G agarose, immobilzed protein, Roche) were added to the lysates, which were further incubated for 4 h at 4°C. Beads were then washed twice with lysis buffer, once with DOC buffer (10 mM Tris-Cl, pH 8, 250 mM LiCl, 0.5% NP-40, 0.5% DOC, 1 mM EDTA, pH 8) and twice with 1 × TE (Tris-Cl 10 mM, EDTA 1 mM, pH 8). Finally, immunoprecipitated complexes were eluted by adding TES buffer (Tris-Cl 50 mM, EDTA 10 mM, 1% SDS, pH 8). Reverse cross-linking was performed by incubating samples overnight at 65°C. Then, samples were treated with 0.2 μg/ml RNase A (Sigma-Aldrich) for 2 h at room temperature and 0.2 μg/ml Proteinase K (Sigma-Aldrich) was added prior incubation for 2 h at 55°C. Extraction of DNA was performed using phenol:chloroform:isoamyl alcohol (25:24:1, Sigma-Aldrich) and precipitated with ethanol supplemented with 5 M NaCl and 1 μl of LPA (Linear PolyAcrylamide, GenElute-LPA, stock: 25 mg/ml, Sigma-Aldrich, Germany). Precipitated DNA was analyzed by quantitative Real-Time PCR.

### Real-time PCR

Total RNA was isolated from yeast cells using the RiboPure Yeast Kit (Applied Biosystems, Ambion, Inc., USA) according to the manufacturer's instructions. RNA was then transcribed to cDNA using the SuperScript II Double-Stranded cDNA Synthesis Kit (Invitrogen, USA) according to the manufacturer's instructions. The quantification of PCR products was performed using the fluorescent dye SYBR Green (Applied Biosystems) and a Real-Time PCR machine (Applied Biosystems, 7900 HT Real-Time PCR System). Oligonucleotides for open reading frames of *TSA1*, *ACT1*, *CLB2*, and *CLB2* promoter were used in this study (see Table 2).

## Results

### Fkh1 and Fkh2 associate with Sir2

**Figure 1:**
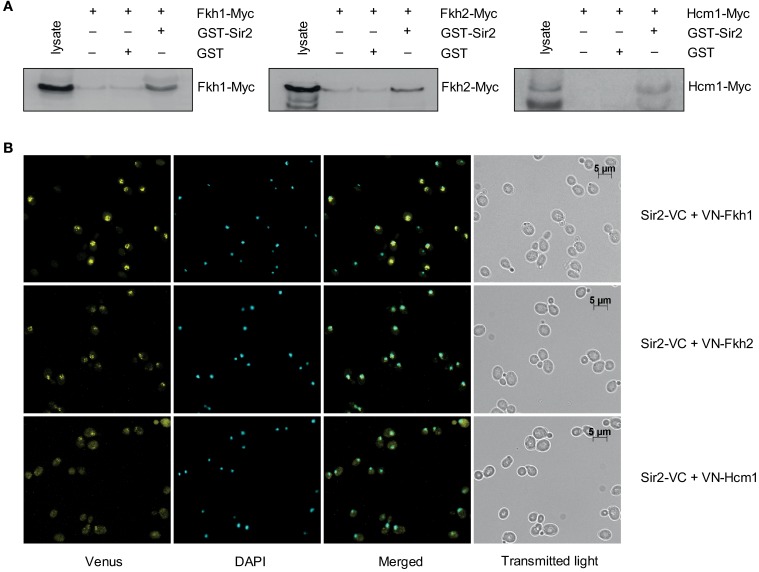
**Fkh1 and Fkh2 associate with Sir2. (A)** Pull-down assay. GST and GST-Sir2 proteins expressed in *E. coli* were immobilized on glutathione sepharose beads and incubated with lysates derived from yeast strains carrying Myc-tagged *FKH1*, *FKH2*, and *HCM1*. Immunodetection of co-precipitated Fkh1-Myc, Fkh2-Myc and Hcm1-Myc was performed with a mouse α-Myc antibody. **(B)** BiFC analysis. Yeast cells expressing the fusion protein Sir2-VC were transformed with plasmids encoding the fusion proteins VN-Fkh1, VN-Fkh2, and VN-Hcm1 under the control of the constitutive GPD promoter. Venus signals were analyzed.

An interaction between the yeast Fkh transcription factor Hcm1 and Sir2 was recently discovered (Rodriguez-Colman et al., 2010). Moreover, it was shown that the nuclear localization of Hcm1 at the G1/S transition is dependent on Sir2 activity, suggesting a Sir2-dependent role in cell cycle regulation. Of note, another member of this family, Fkh1, was found to play a role in Sir2-dependent silencing at the mating-type locus *HMR* (Hollenhorst et al., 2000), suggesting a potential interplay between Fkh1/Fkh2 and Sir2 as well (see network illustration in Figure 8 for the known relationship among Fkh1/Fkh2, Hcm1, and Sir2). To explore this in more detail, we first investigated whether Fkh1 and Fkh2 are also found in association with Sir2 performing GST pull-down assays. GST and GST-Sir2 proteins were expressed and purified from *E. coli* and immobilized on glutathione sepharose beads, which were splitted into three aliquots. Then, each sample was incubated with lysates prepared from yeast strains expressing Myc-tagged Fkh1, Fkh2, or Hcm1, the latter being used as control. As shown in Figure 1A (left and middle panels), we were able to precipitate Fkh1-Myc and Fkh2-Myc with GST-Sir2. Only a minimal amount was detected in samples with sepharose beads alone or with GST-coupled resins, indicating an interaction between Fkh1/Fkh2 and Sir2. In addition, we were also able to confirm the previously described interaction between Hcm1 and Sir2 (Figure 1A, right panel). Since higher protein levels for Fkh1 and Fkh2 have been detected *in vivo* as compared to Hcm1 (Rodriguez-Colman et al., 2010), the GST pull-down assay may reflect this finding, as equal concentrations of protein lysates were loaded as input.

To further verify the observed association between Fkh1/Fkh2 and Sir2, we carried out a BiFC analysis as described in Materials and Methods. Yeast cells expressing Sir2-VC were transformed with either plasmids p426GPD-VN-Fkh1, p426GPD-VN-Fkh2, or p426GPD-VN-Hcm1 encoding fusion proteins between the N-terminal region of Venus and the C-terminal region of the selected Fkh transcription factors. Mid-logarithmic cultures of isolated transformants were fixed with ethanol, nuclei were stained with DAPI, and fluorescent BiFC signals were monitored by microscopy. This analysis revealed that yeast cells expressing Sir2-VC/VN-Fkh1 (Figure 1B, upper panel), Sir2-VC/VN-Fkh2 (Figure 1B, middle panel), and Sir2-VC/VN-Hcm1 (Figure 1B, bottom panel) exhibited nuclear BiFC signals. Moreover, fluorescence signal intensities appeared to be different among the analyzed interaction pairs. In particular, cells co-expressing Sir2-VC and VN-Fkh1 showed the strongest BiFC signal, followed by cells co-expressing Sir2-VC/VN-Fkh2 and cells co-expressing Sir2-VC/VN-Hcm1, probably again reflecting different post-transcriptional regulation of the Fkh transcription factors *in vivo*, as reported (Rodriguez-Colman et al., 2010). Thus, these analyses revealed that nuclear Fkh1 and Fkh2 interact with Sir2.

### Fkh1 and Fkh2 act in concert with Sir2 to repress gene transcription

**Figure 2:**
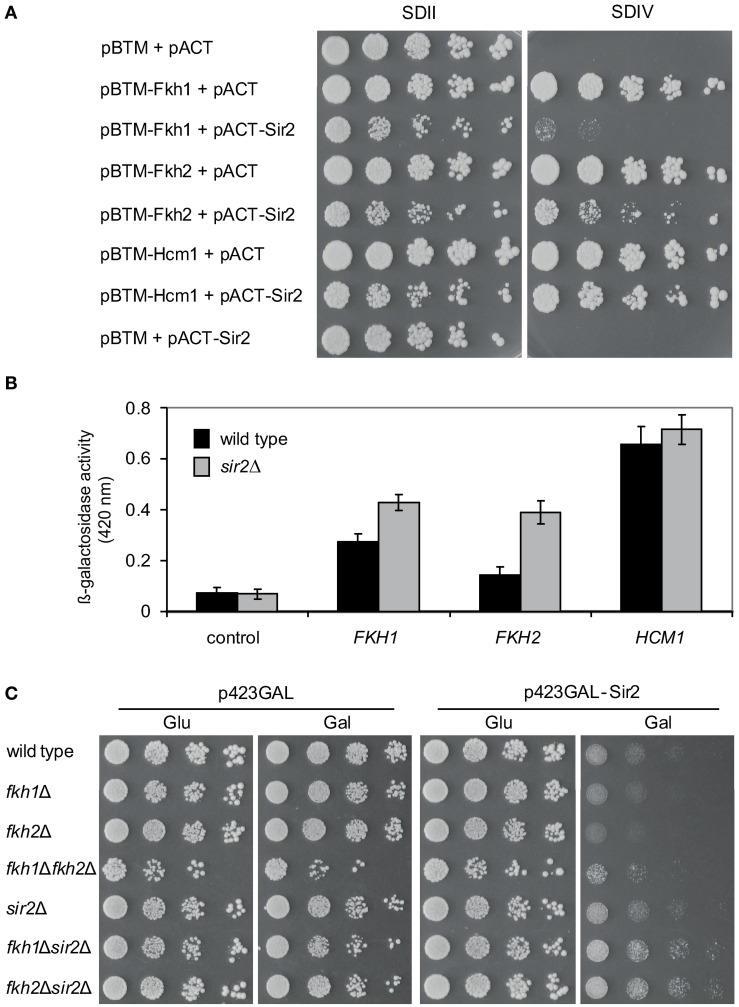
**Sir2 represses gene transcription via Fkh transcription factors. (A)** Reporter gene activity assay. Yeast cultures were spotted in 1:5 serial dilutions onto SDII and SDIV media, and cell growth was analyzed after 5 days. **(B)** Liquid β-galactosidase assay. Protein lysates were prepared from exponentially growing yeast cells expressing LexA-Fkh1, LexA-Fkh2, or LexA-Hcm1 fusion proteins. Each bar represents the mean average obtained from three independent experiments. **(C)** Genetic interaction studies. Wild type strain BY4741 and deletion strains *fkh1*Δ, *fkh2*Δ, *sir2*Δ, *fkh1*Δ*sir2*Δ, and *fkh2*Δ*sir2*Δ were transformed with p423GAL -Sir2 or vector p423GAL as control. Subsequently, yeast cells were grown to mid-exponential phase, spotted in 1:5 serial dilutions on glucose or on galactose plates, and growth of yeast cells was analyzed after 3 days. The assay was performed three times, and one representative experiment is shown.

As aforementioned, Fkh1 plays a role in Sir2-dependent silencing at the mating-type locus HMR, suggesting a potential involvement of Sir2 in transcriptional silencing via Fkh proteins (Hollenhorst et al., 2000). Moreover, both Fkh1 and Fkh2 are involved in the recruitment of chromatin remodeling factors leading to the repression of their target genes (Sherriff et al., 2007). In order to investigate whether Sir2 is involved in the repression of Fkh1/Fkh2-dependent genes, we performed a reporter gene activity assay, which is a modified Y2H assay. In the course of protein-protein interaction studies we discovered that the expression of LexA-tagged Fkh1, Fkh2 as well as Hcm1 *per se* led to reporter gene activity allowing yeast cells to grow on the respective selective medium (data not shown and Figure 2A). As a result, this finding allows analyzing whether co-expression of Sir2 has a repressive effect on this observed reporter gene activity, since in this case growth of yeast cells on the respective selective medium is expected to be reduced or inhibited. L40ccua yeast cells were co-transformed with plasmids pBTM-Fkh1 and pACT-Sir2, pBTM-Fkh2 and pACT-Sir2, or pBTM-Hcm1 and pACT-Sir2, respectively. Co-transformation of empty Y2H plasmids, or combination of the used bait and prey constructs and empty Y2H plasmids were used as controls. Then, transformants were selected and spotted in 1:5 serial dilutions on selective media and cell growth was monitored after 5 days. As shown in Figure 2A, yeast cells expressing the fusion proteins LexA-Fkh1, LexA-Fkh2, or LexA-Hcm1 in combination with the activation domain (AD) alone exhibited strong growth on selective SDIV medium, indicating auto-activation of reporter genes, as expected from our earlier findings (unpublished data). The growth of yeast cells co-expressing LexA-Fkh1 and AD-Sir2 or LexA-Fkh2 and AD-Sir2 was strongly reduced on SDIV medium, indicating a decreased reporter gene activity. Interestingly, cells expressing LexA-Hcm1 and AD-Sir2 exhibited only a slight growth reduction on SDIV medium, suggesting a lower Sir2-dependent repression of reporter gene activity under these conditions. Nevertheless, these observed growth differences of cells expressing LexA-Fkh1/AD-Sir2, LexA-Fkh2/AD-Sir2, or LexA-Hcm1/AD-Sir2 could also reflect the different post-transcriptional regulation of the Fkh transcription factors (Rodriguez-Colman et al., 2010). Following this line, the observed reduction in reporter gene activity was more severe for cells co-expressing LexA-Fkh1 and AD-Sir2 as compared to cells co-expressing LexA-Fkh2 and AD-Sir2. These results demonstrated that Sir2 expression impairs cell growth on SDIV medium by repressing autoactivity of reporter genes via its association with Fkh1 and Fkh2.

In order to further verify the observed Sir2-mediated repression of reporter gene activity in the presence of LexA-Fkh1/Fkh2/Hcm1, we investigated the effect of *SIR2* deficiency in this modified Y2H approach. L40ccua and L40ccua/*sir2*Δ cells were transformed with the respective plasmids encoding LexA-Fkh1, LexA-Fkh2, LexA-Hcm1, or the corresponding control vectors. Then, transformants were selected, cultured to mid-logarithmic phase in liquid SDII medium, and β-galactosidase activity was measured as described in Materials and Methods. This analysis revealed that *lacZ* reporter gene activity was significantly enhanced in *sir2*Δ cells expressing LexA-Fkh1 or Lex-Fkh2 as compared to wild type cells (Figure 2B). Interestingly, wild type and *sir2*Δ cells expressing LexA-Hcm1 exhibited a similar β-galactosidase activity as compared to wild type cells, suggesting a minimal Sir2-dependent repression of the *lacZ* reporter gene activity. In sum, these data demonstrate that overexpression of Fkh1 and Fkh2 in *sir2*Δ strain represses beta-galactosidase reporter gene activity to a less extent compared to wild type.

Of note, this approach also revealed that the growth of yeast cells co-expressing LexA-Fkh1 and AD-Sir2, LexA-Fkh2 and AD-Sir2, or LexA-Hcm1 and AD-Sir2 was reduced on SDII medium—which is only selective for the presence of plasmids—as compared to cells expressing the Fkh proteins alone (Figure 2A). Importantly, a reduced colony size was not observed in cells expressing only AD-Sir2, supporting the functional relationship between Sir2 and Fkh1 and Fkh2.

To further validate the observed functional relationship between Fkh1/Fkh2 and Sir2 on a different cellular level, we performed additional genetic analyses. We generated a number of single and double deletion strains such as *fkh1*Δ, *fkh2*Δ, *fkh1*Δ*fkh2*Δ, *sir2*Δ, *fkh1*Δ*sir2*Δ, and *fkh2*Δ*sir2*Δ. Interestingly, the triple deletion of *FKH1*, *FKH2*, and *SIR2* was not viable (data not shown), suggesting a genetic interplay between these proteins. We transformed these respective deletions strains with plasmid p423GAL-Sir2, which carries *SIR2* under the control of a galactose inducible promoter, or with plasmid p423GAL as control. After selection of transformants, these were grown in liquid media to logarithmic phase (OD_600_ ~0.6), spotted in 1:5 serial dilutions on medium supplemented with glucose (control) or galactose (to induce expression of Sir2), and growth of yeast cells was analyzed after 5 days. As illustrated in Figure 2C, growth of wild type cells overexpressing Sir2 was reduced compared to cells carrying the empty vector. Interestingly, *fkh1*Δ and especially *fkh2*Δ cells overexpressing Sir2 also exhibited a reduced growth compared to cells carrying the empty vector. This growth reduction was slightly stronger compared to wild type cells overexpressing Sir2, again suggesting a functional interplay between Fkh transcription factors and Sir2. In comparison to single gene deletions and wild type strains, deletion of both *FKH1* and *FKH2* rescues Sir2-dependent growth defects, indicating that Sir2 activity is dependent upon the presence of Fkh1 and Fkh2. Of note, only a slight reduction in growth was observed for *fkh1*Δ*sir2*Δ and *fkh2*Δ*sir2*Δ cells overexpressing Sir2 compared to the control, demonstrating that Sir2 deficiency rescues growth reduction of cells lacking either *FKH1* or *FKH2*. Taken together, these findings provide evidence that Sir2 overexpression affects growth of yeast cells likely through global repression of genes regulating cell growth.

### Fkh1/Fkh2 and Sir2 control CLB2 transcription

**Figure 3:**
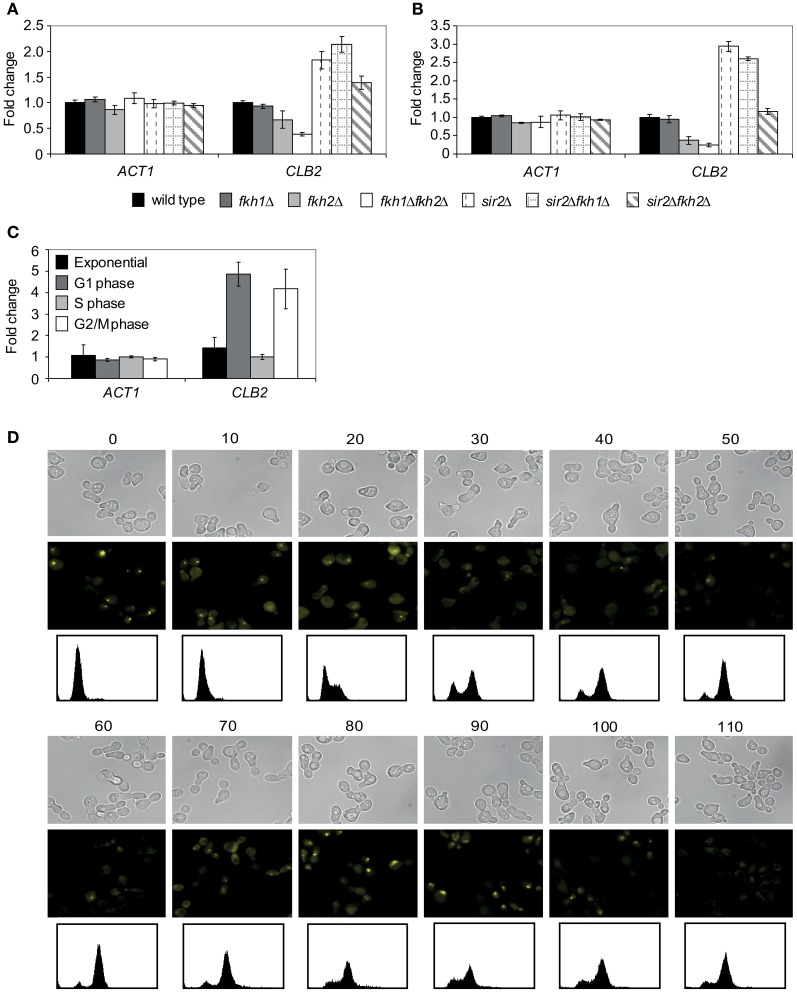
**Sir2 regulates the *CLB2* transcript level and binds to the *CLB2* promoter in a cell cycle-dependent manner. (A,B)** Quantitative Real-Time PCR. Total RNA was isolated from **(A)** hydroxyurea- or **(B)** nocodazole- arrested wild type, *fkh1*Δ, *fkh2*Δ, *fkh1*Δ*fkh2*Δ, *sir2*Δ, *fkh1*Δ*sir2*Δ, and *fkh2*Δ*sir2*Δ cells. The *ACT1* gene was used as control. Each bar represents the mean average obtained from three independent experiments. **(C)** ChIP assay. Protein/DNA complexes were precipitated from cells grown in exponential phase or synchronized with α-factor (G1 phase), hydroxyurea (S phase) or nocodazole (M phase) using an anti-Myc antibody. Each bar represents the mean average obtained from three independent experiments. **(D)** BiFC analysis. Haploid cells expressing the fusion protein Sir2-VC were transformed with plasmid p426GPD-VN-Fkh1, and selected transformants were synchronized in exponential growth (OD_600_ ~0.6) with α-factor. Arrested cells were released into fresh media and samples were collected every 10 min for the detection of BiFC signals by fluorescence microscopy. DNA content of samples was determined by propidium iodide staining and FACS analysis.

Since Fkh1 and Fkh2 are involved in the activation of G2/M cluster genes driving cell division, we investigated the role of Sir2 in the regulation of *CLB2* transcription. Previous data have shown that Fkh2 directly recruits the HDAC complex Sin3/Rdp3 silencing the *CLB2* promoter in M/G1 phase (Hollenhorst et al., 2000; Ho et al., 2002), whereas Fkh2 activates *CLB2* cluster genes in S and G2/M phases (Loy et al., 1999; Koranda et al., 2000; Hollenhorst et al., 2001). Moreover, transcriptional analyses revealed that deletion of *FKH1* enhances *CLB2* transcription, whereas overexpression of *FKH1* reduces *CLB2* transcription (Hollenhorst et al., 2000). Since transcriptional activation of *CLB2* cluster genes occurs in S and G2/M phase, we first investigated whether the *CLB2* mRNA level was altered in *fkh1*Δ, *fkh2*Δ, *fkh1*Δ*fkh2*Δ, *sir2*Δ, *fkh1*Δ*sir2*Δ, and *fkh2*Δ*sir2*Δ strains by arresting cells with hydroxyurea or nocodazole, respectively. The quantitative Real-Time PCR analyses of S phase arrested cells revealed an enhanced *CLB2* transcript level in *sir2*Δ, *fkh1*Δ*sir2*Δ, and *fkh2*Δ*sir2*Δ deletions compared to wild type strain (Figure 3A). Cells lacking *FKH2*, but not *FKH1*, showed a decreased *CLB2* transcript level compared to wild type cells, consistent with the known function of Fkh2 in the activation of *CLB2* cluster genes (Loy et al., 1999; Koranda et al., 2000; Hollenhorst et al., 2001). As reported (Zhu et al., 2000; Hollenhorst et al., 2001), simultaneous disruption of both *FKH1* and *FKH2* severely reduces *CLB2* mRNA transcript level (Figure 3A). *Sir2*Δ and *fkh1*Δ*sir2*Δ cells arrested in G2/M phase showed a strong enrichment in *CLB2* transcript level, whereas only a slightly higher *CLB2* transcript level was detected in *fkh2*Δ*sir2*Δ cells (Figure 3B). In comparison to wild type, a decrease in *CLB2* transcription was observed for *fkh2*Δ cells as well as for *fkh2*Δ*sir2*Δ cells compared to *sir2*Δ cells. Thus, Sir2 is involved in the repression of *CLB2* transcription with higher impact in G2/M phase.

In order to address the repressive function of Sir2 in the Fkh-mediated regulation of *CLB2* in more detail, we investigated whether Sir2 is directly bound to the *CLB2* promoter by performing ChIP assays. Yeast cells expressing endogenous Sir2-Myc were cultured to mid-exponential phase and growth arrest was performed by adding α-factor (G1 phase), hydroxyurea (S phase) or nocodazole (G2/M phase). Then, Sir2-Myc protein was precipitated with an epitope-specific antibody, and co-precipitated DNA fragments were analyzed with quantitative Real-Time PCR using *CLB2* promoter-specific oligonucleotides. We observed a weak enrichment of *CLB2* promoter-specific DNA in exponentially growing cells, indicating that binding of Sir2 is low (Figure 3C), whereas a strong enrichment was detected in cells arrested in G1 and G2/M phases, indicating stronger binding of Sir2 to the *CLB2* promoter. No enrichment of *CLB2* promotor-specific DNA was observed in cells arrested in S phase. In conclusion, the different *CLB2* promoter occupancy of Sir2 suggests an association between Sir2 and Fkh transcription factors in G1 and G2/M phase, which is consistent with higher *CLB2* transcript levels detected in these cell cycle phases (Figure 3B).

Finally, this finding prompted us to further investigate whether the association between Fkh transcription factors and Sir2 is cell cycle-dependent. Cells co-expressing Venus fusion proteins Sir2-VC and VN-Fkh1 were synchronized in G1 phase with α-factor, and the presence of BiFC signals was monitored at different stages of the cell cycle (Figure 3D). In agreement with our ChIP assays, BiFC signals were observed in G1 phase (0–20 min). After 20 min, the BiFC signal disappeared in correspondence to the transcription of *CLB2* cluster genes during late S phase (Breeden, 2000; Futcher, 2000). The BiFC signal was absent until late M phase (70 min), but it raised again until the subsequent G1 phase (80–90 min). Thus, the association between Fkh1 and Sir2 oscillates throughout the cell cycle and correlates with the transcriptional inactivation of *CLB2* at the M/G1 transition.

### The functional interplay between Fkh1/Fkh2 and Sir2 is stress responsive

**Figure 4:**
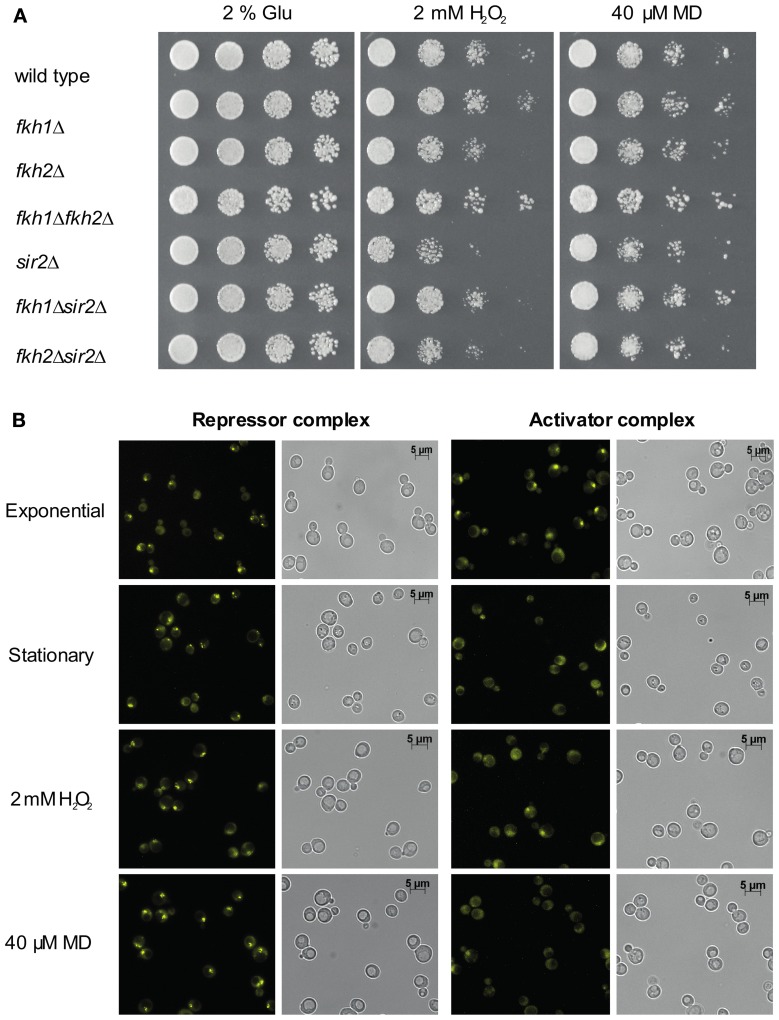
**Interplay between Fkh transcription factors and Sir2 under oxidative stress conditions. (A)** Growth analysis. Wild type, *fkh1*Δ, *fkh2*Δ, *fkh1*Δ*fkh2*Δ, *sir2*Δ, *fkh1*Δ*sir2*Δ, and *fkh2*Δ*sir2*Δ deletion strains were grown in YPD medium to saturation overnight and spotted in 1:5 serial dilutions on CSM medium containing 2 mM H_2_O_2_ or 40 μM MD. Cell growth was analyzed after 3 days. The assay was performed three times, and one representative experiment is shown. **(B)** BiFC analysis. Haploid cells expressing Sir2-VC or Ndd1-VC were transformed with plasmid p426GPD-VN-Fkh1 or p426GPD-VN-Fkh2, respectively. Subsequently, yeast cells were analyzed for BiFC signals in exponential phase (OD_600_ ~0.6), in stationary phase (OD_600_ ~1.6) and in the presence of 2 mM H_2_O_2_ or 40 μM MD.

Since Hcm1 is involved in the activation of genes that regulate oxidative stress resistance (Rodriguez-Colman et al., 2010), and induction of oxidative stress by H_2_O_2_ and MD resulted in Fkh-dependent cell cycle arrest (Shapira et al., 2004), we investigated the functional interplay between Fkh proteins and Sir2 under such stress conditions. First, a genetic analysis was performed with wild type, *fkh1*Δ, *fkh2*Δ, *fkh1*Δ*fkh2*Δ, *sir2*Δ, *fkh1*Δ*sir2*Δ, and *fkh2*Δ*sir2*Δ cells that were grown overnight to saturation and spotted on SC medium supplemented with 2 mM H_2_O_2_—which delays cell growth in S phase followed by G2/M arrest—or 40 μM MD—which arrests cells in G1 phase (Flattery-O'Brien and Dawes, 1998; Shapira et al., 2004). As shown in Figure 4A, a reduced growth of all yeast strains was observed in the presence of H_2_O_2_ or MD. *fkh2*Δ, *sir2*Δ, *fkh1*Δ*sir2*Δ, and *fkh2*Δ*sir2*Δ cells exhibited a reduced growth in the presence of H_2_O_2_ and MD as compared to wild type cells, with stronger defects observed for *sir2*Δ strain. However, *fkh1*Δ*fkh2*Δ cells did not show any growth inhibition on plates supplemented with H_2_O_2_ or MD. Of note, *sir2*Δ cells showed reduction of growth in the presence of oxidants, which was not detected for *fkh1*Δ*sir2*Δ and *fkh2*Δ*sir2*Δ cells. Thus, these results suggest that Fkh1 and Fkh2 are also important for Sir2 function in response to oxidative stress.

To further support this finding, we treated cells co-expressing the Venus fusion proteins Sir2-VC and VN-Fkh1 with H_2_O_2_ or MD. Since deletion of both *FKH1* and *FKH2* impede normal lifespan and stress resistance of yeast cells particularly in stationary phase (Postnikoff et al., 2012), we also analyzed cells grown to stationary phase. As shown in Figure 4B, strong BiFC signals were observed in the majority of cells treated with H_2_O_2_ or MD. Moreover, fluorescent signals were observed in nearly all cells in stationary phase as compared to logarithmic growing cells, indicating a stress responsiveness of the Fkh/Sir2 interaction. This finding indicates that the Fkh/Sir2 complex could repress *CLB2* transcription under such conditions, suggesting that the amount of activator complexes of *CLB2* transcription might be reduced. Consequently, we further analyzed whether a known activator complex of *CLB2* transcription may show antagonistic appearance. Here, we used the described complex between Ndd1 and Fkh2 driving periodic expression of genes required for G2/M transitions of the cell cycle (Darieva et al., 2003; Reynolds et al., 2003; Pic-Taylor et al., 2004). As shown in Figure 4B, BiFC signals indicating the activator complex were observed in exponentially growing cells, but were not detectable in stationary cells or reduced in cells treated with H_2_O_2_ or MD. This indeed demonstrates antagonistic appearance between a repressor and activator complex of the *CLB2* transcription in stationary phase and under oxidative stress conditions.

### Cellular stress affects binding of Sir2 to the CLB2 promoter and Clb2 expression

**Figure 5:**
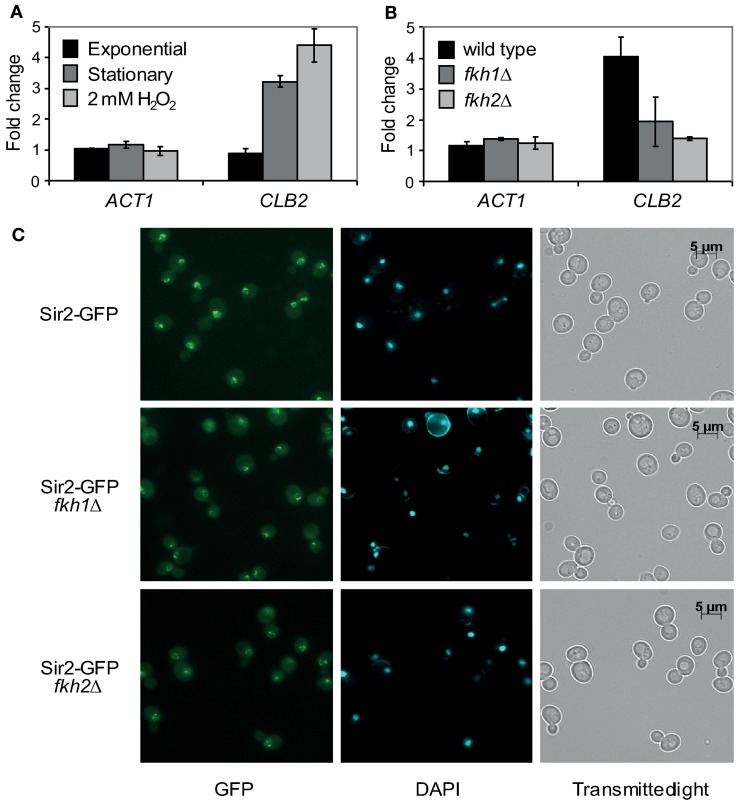
**Sir2 binds to the *CLB2* promoter via Fkh1 and Fkh2 under stress conditions. (A,B)** ChIP assay. **(A)** Haploid cells were grown to exponential phase (OD_600_ ~0.6), stationary phase (OD_600_ ~1.6) or treated with 2 mM H_2_O_2_. Protein extracts were prepared and ChIP experiments were carried out using an anti-Myc antibody. The *ACT1* gene was used as control. Each bar represents the mean average obtained from three independent experiments. **(B)** Haploid wild type and *fkh1*Δ or *fkh2*Δ cells expressing a C-terminal tagged Sir2-Myc fusion protein were grown to stationary phase (OD_600_ ~1.6), and ChIP experiments were performed using an anti-Myc antibody. The *ACT1* gene was used as control. Each bar represents the mean average obtained from three independent experiments. **(C)** Localization studies. Haploid wild type, *fkh1*Δ and *fkh2*Δ cells expressing GFP-tagged Sir2 were grown overnight to saturation (OD_600_ ~1.6), and Sir2-GFP signals were analyzed by fluorescence microscopy.

The ubiquitous BiFC signal observed in cells grown to stationary phase or treated with oxidants suggests that the Fkh/Sir2 association might be strengthened under such conditions. Therefore, we investigated whether an enhanced Sir2 occupancy at the *CLB2* promoter occurs. To address this question, ChIP assays with cells endogenously expressing Myc-tagged Sir2 were carried out in exponential phase (OD_600_ ~0.6), stationary phase (OD_600_ ~1.6) or upon H_2_O_2_ treatment. Immunoprecipitation assays were performed and enrichment of Sir2 at the *CLB2* gene promoter was quantified by Real-Time PCR as described. Higher amount of *CLB2* promoter-specific DNA was detected in cells grown to stationary phase and those treated with H_2_O_2_, as compared to exponentially growing cells (Figure 5A). This result indicates an enhanced binding of Sir2 to the *CLB2* promoter in response to stress conditions, consistent with stronger Fkh/Sir2 BiFC signals observed in stationary phase and oxidant-treated cells (Figure 4B). To further substantiate these findings, we additionally investigated the binding of Sir2 to the *CLB2* promoter in the absence of *FKH1* and *FKH2*. *Fkh1*Δ and *fkh2*Δ deletion mutants carrying chromosomal Myc-tagged *SIR2* were grown to saturation (OD_600_ ~1.6) and used in ChIP assays. In comparison to wild type, enrichment of Sir2 at the *CLB2* promoter was decreased for cells lacking either *FKH1* or *FKH2* (Figure 5B), indicating that Fkh1 and Fkh2 are necessary for the binding of Sir2 to the *CLB2* promoter. In order to explore whether the nuclear localization of Sir2 is dependent on the presence of Fkh1 and Fkh2, we also investigated the localization of Sir2-GFP in *FKH1* and *FKH2* deficient cells. Indeed, the intensity of the Sir2-GFP signal was reduced in *fkh1*Δ and *fkh2*Δ cells (Figure 5C), thus suggesting that Fkh1 and Fkh2 are potentially required for the nuclear localization of Sir2 and its binding to the *CLB2* promoter.

**Figure 6:**
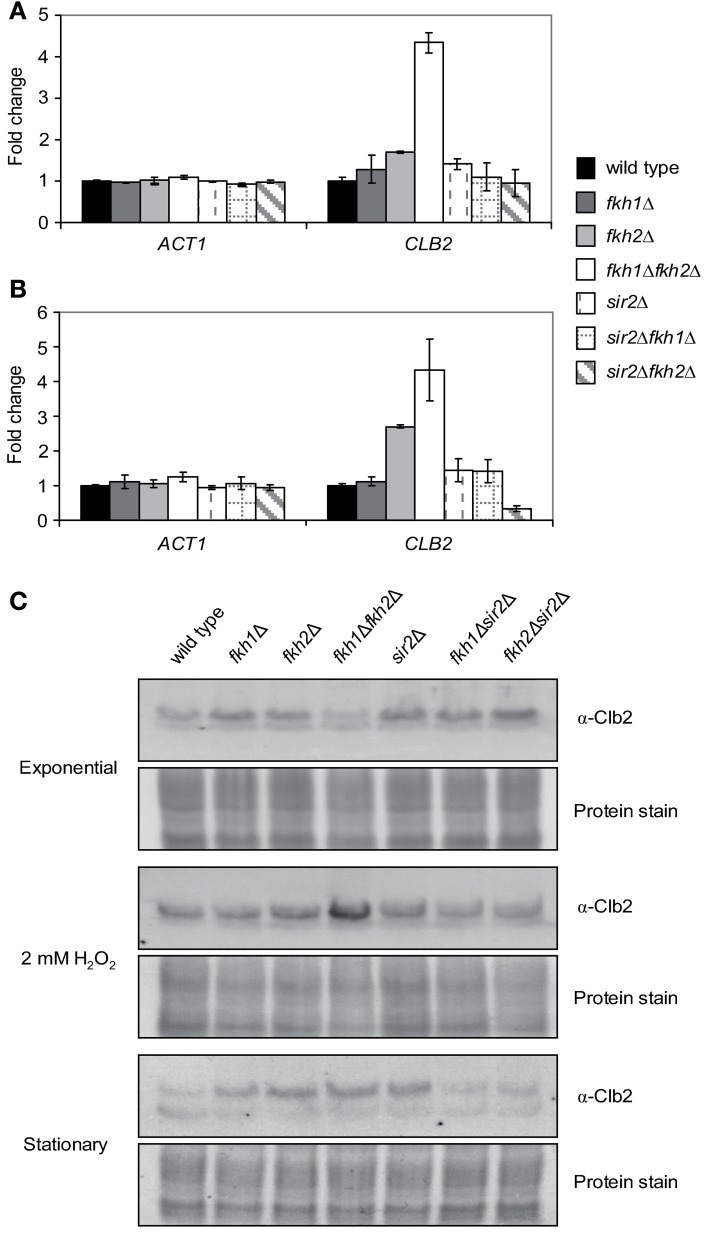
***CLB2* transcript and protein levels are altered under stress conditions. (A,B)** Quantitative Real-Time PCR. Total RNA was prepared from exponentially growing wild type and *fkh1*Δ, *fkh2*Δ, *fkh1*Δ*fkh2*Δ, *sir2*Δ, *fkh1*Δ*sir2*Δ, and *fkh2*Δ*sir2*Δ cells (OD_600_ ~0.6) that were treated with 2 mM H_2_O_2_ for 90 min **(A)** or grown to stationary phase (OD_600_ ~1.6) **(B)**. The *ACT1* gene was used as control. Each bar represents the mean average obtained from three independent experiments. **(C)** Protein extracts were isolated from exponential growing cells (OD_600_ ~0.6), cells incubated with 2 mM H_2_O_2_ for 90 min or cells grown to stationary phase. Clb2 levels were determined by Western Blot using an α-Clb2 specific antibody. Coomassie Brilliant Blue protein staining was used as a loading control.

Given the significant binding of Sir2 to the *CLB2* promoter under stress conditions, we investigated whether this finding correlates with *CLB2* transcript levels and Clb2 protein levels in the respective deletion strains. For the transcriptional analysis, wild type, *fkh1*Δ, *fkh2*Δ, *fkh1*Δ*fkh2*Δ, *sir2*Δ, *fkh1*Δ*sir2*Δ, and *fkh2*Δ*sir2*Δ strains were incubated overnight (OD_600_ ~1.6) or treated with H_2_O_2_ in the exponential growth phase (OD_600_ ~0.6). Total RNA was then extracted from wild type and deletion strains, transcribed to cDNA, and *CLB2* transcript levels were quantified by Real-Time PCR. A strong increase in *CLB2* transcript was observed for *fkh1*Δ*fkh2*Δ cells treated with H_2_O_2_ in comparison to wild type cells (Figure 6A). Moreover, *CLB2* transcripts were slightly increased in *sir2*Δ, *fkh1*Δ, and *fkh2*Δ deletion mutants, whereas no significant alteration was observed in *fkh1*Δ*sir2*Δ and *fkh2*Δ*sir2*Δ mutants. Interestingly, same observations were made in case of stationary cells. A significant increase in *CLB2* transcripts was detected for *fkh2*Δ and *fkh1*Δ*fkh2*Δ cells as compared to wild type cells (Figure 6B). Consistently, increased *CLB2* transcript levels were observed in *fkh1*Δ*fkh2*Δ cells and H_2_O_2_-treated cells. Moreover, a slight enrichment was detected in *fkh1*Δ*sir2*Δ cells, but not in *fkh2*Δ*sir2*Δ cells (Figure 6B). These findings are in agreement with the protein levels observed. In fact, as shown in Figure 6C, Clb2 levels were highest in *fkh1*Δ*fkh2*Δ cells after treatment with H_2_O_2_, supporting the fact that both transcription factors are involved in the repression of the *CLB2* promoter. Only a moderate increase in Clb2 level was observed in *fkh2*Δ and *sir2*Δ cells in comparison to wild type cells. No alteration in Clb2 level was observed in *fkh1*Δ cells; however the level in *fkh1*Δ*sir2*Δ and *fkh2*Δ*sir2*Δ cells was slightly lower as compared to *sir2*Δ cells. Of note, similar results were obtained for cells grown to stationary phase (Figure 6C). In this condition, a strong increase in Clb2 level was observed for *fkh2*Δ, *sir2*Δ, and *fkh1*Δ*fkh2*Δ cells as compared to wild type cells, whereas a slight increase was observed for the *fkh1*Δ strain. In conclusion, these results indicate a direct involvement of Sir2 in the regulation of *CLB2* transcription mediated by Fkh1 and Fkh2 under stress conditions. However, since *fkh2*Δ and *sir2*Δ cells showed generally a slight increase in *CLB2* levels, an involvement of other co-regulators should be taken into consideration.

### High Clb2 levels affect cell survival under oxidative stress conditions

**Figure 7:**
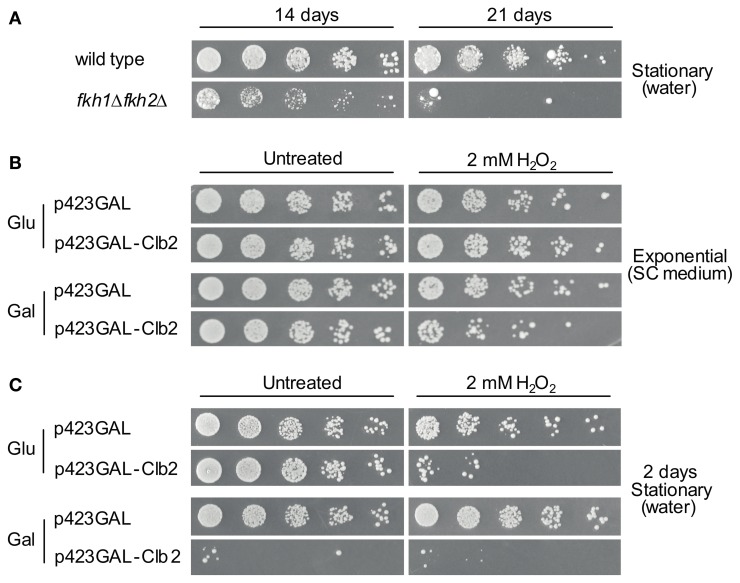
**High Clb2 levels influence survival of yeast cells under oxidative stress conditions. (A)** Survival of wild type and *fkh1*Δ*fkh2*Δ cells upon nutrient limitation. Yeast strains were grown to saturation (OD_600_ ~1.6) and further incubated in H_2_O. Samples of cultures were spotted in 1:5 serial dilutions on SC medium after 14 or 21 days. **(B,C)** Clb2 overexpression in exponentially grown **(B)** and stationary **(C)** cells in presence of H_2_O_2_. Yeast cells transformed with plasmids p423GAL-Clb2 or p423GAL were incubated in SC medium containing 2% galactose with or without 2 mM H_2_O_2_ for 12 h. Yeast cultures were then spotted in 1:5 serial dilutions on glucose- or galactose-containing plates, and growth was analysed after 3 days. One representative experiment is shown.

A substantial reduction in chronological life span under caloric restriction and an enhanced cytotoxicity in presence of H_2_O_2_ has been observed for yeast cells lacking *FKH1*/*FKH2* (Postnikoff et al., 2012). In the first step, we confirmed that the *fkh1*Δ*fkh2*Δ strain used in our study exhibits reduction in life span under this condition. For this purpose, life span of *fkh1*Δ*fkh2*Δ cells was investigated as described in Materials and Methods. As shown in Figure 7A, growth reduction was monitored for *fkh1*Δ*fkh2*Δ cells grown after 2 weeks in comparison to wild type cells. After 3 weeks, a severe decline in cell viability of *fkh1*Δ*fkh2*Δ cells was observed, in agreement with the previous finding (Postnikoff et al., 2012). Subsequently, we investigated whether the higher Clb2 level observed in *fkh1*Δ*fkh2*Δ cells after treatment with 2 mM H_2_O_2_ might be important for cell survival under oxidative stress. For this purpose, wild type cells were transformed with plasmid p423GAL-Clb2, or with plasmid p423GAL as control. After selection of transformants, overnight cultures were diluted to an OD_600_ ~0.3. Then, cultures were further incubated in galactose-containing SC medium supplemented with 2 mM of H_2_O_2_. As control, cells were left untreated. As shown in Figure 7B, H_2_O_2_-treated cells overexpressing Clb2 showed a strong reduction in growth compared to control cells carrying the empty vector, indicating that a tight control of *CLB2* transcription is mandatory to maintain cell survival under oxidative stress conditions. Since enhanced Clb2 levels were also detected in stationary *fkh1*Δ*fkh2*Δ cells, the effect of enhanced Clb2 levels on survival of stationary cells under oxidative stress was finally analysed. Overnight cultures of the respective strains were splitted and incubated in galactose- or glucose-containing SC medium for 12 h. Then, cells were washed, resuspended in water with or without 2 mM H_2_O_2_, incubated for additional 48 h, and spotted in serial dilutions on selective SC medium containing 2% glucose. Interestingly, Clb2 overexpression in stationary cells caused a severe loss of cell viability under oxidative stress conditions (Figure 7C), which was more drastic compared to cells grown exponentially. The same observation hold for H_2_O_2_-treated cells carrying plasmid p423GAL-Clb2 under non-induced conditions, which might reflect the known basal transcriptional activity of the galactose promoter (Turner et al., 2010). Striking, Clb2 overexpression already resulted in a severe reduction in viability of stationary cells, suggesting that control of Clb2 expression is not only crucial for survival of yeast cells exposed to oxidative stress, but also for those entering post-mitotic stages.

## Discussion

**Figure 8:**
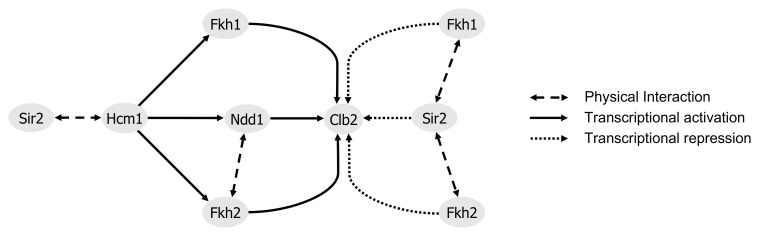
**Regulatory model of Clb2 expression.** The transcription factor Hcm1 is required for periodic expression of the G2/M phase-specific regulators Fkh1, Fkh2, and Ndd1 (Pramila et al., 2006). Control of Clb2 expression requires the binding of Fkh1, Fkh2, and the transcriptional coactivator Ndd1 (Darieva et al., 2003; Reynolds et al., 2003; Pic-Taylor et al., 2004). In late M phase and during G1 phase, Ndd1 depleted from *CLB2* promoter allows for binding of the HDAC Sir2. Our findings suggest a repressive association between Fkh1/Fkh2 and Sir2, with a predominant role for Fkh1. This is also supported by findings of (Hollenhorst et al., 2001). In line with our results, nuclear localization of Hcm1 was shown to be Sir2-dependent (Rodriguez-Colman et al., 2010).

The evolutionary conserved Fkh transcription factors play essential roles in many physiological processes, such as control of progression throughout the cell cycle by activating and repressing genes involved in mitotic exit. In fact, when activated, these factors promote expression of the G2/M target genes required for cell division. In budding yeast, Fkh1 and Fkh2 regulate the expression of the mitotic cyclin Clb2, which level is critically required for mitotic exit. It has been proposed that Fkh transcription factors might function to stabilize chromatin-mediated repression or promote other repressing activities, such as the recruitment of HDACs to promoters of target genes (Veis et al., 2007). Although molecular mechanisms regulating *CLB2* expression are believed to be unraveled, evidence indicates that HDACs might interfere with Fkh-dependent regulation of Clb2 (Hollenhorst et al., 2000; Ho et al., 2002). It has been previously shown that expression of Fkh1 at high copy suppresses silencing defects at the *HMRa* locus in *sir1*Δ cells (Hollenhorst et al., 2000; Casey et al., 2008), and that Fkh1 failed to enhance silencing in *clb2*Δ*sir1*Δ cells, suggesting that Fkh1-dependent silencing requires *CLB2* (Casey et al., 2008). The results presented here identify Sir2 as a potential negative regulator of *CLB2* transcription, and Figure 8 shows the major outcome of the present study together with the known relationship among Fkh1/Fkh2, Hcm1, Sir2, and Clb2. Fkh1 and Fkh2 interact with Sir2 *in vitro* and *in vivo*, and act in concert with Fkh1 and Fkh2 to control *CLB2* transcription. Together with the evidence that Sir2 interacts with, and regulates the nuclear localization of Hcm1 (Rodriguez-Colman et al., 2010), our findings suggest that Sir2 and Fkhs facilitate epigenetic regulation of cell cycle genes. We further demonstrated that Sir2 binds to the *CLB2* promoter in G1 and M phase in a Fkh1/Fkh2-dependent manner, therefore supporting the fact that inactivation of *CLB2* occurs during the M/G1 transition. In agreement with this hypothesis, our time course analysis revealed a strong association between Fkh1 and Sir2 in G1 and late M phase. Moreover, *CLB2* promoter occupancy of Sir2 was reduced in both *fkh1*Δ and *fkh2*Δ deletion strains. Consistently, we observed that the deletion of both *FKH1* and *FKH2* leads to a strong increase of *CLB2* transcription in G1 phase (our unpublished data), highlighting their repressive function in regulating G2/M genes. Although both transcription factors were shown to bind the *CLB2* cluster promoters *in vitro*, only Fkh2 binds it *in vivo* (Koranda et al., 2000; Kumar et al., 2000; Pic et al., 2000; Zhu et al., 2000; Hollenhorst et al., 2001; Voth et al., 2007) due to the cooperative binding with the transcription factor Mcm1 (Hollenhorst et al., 2001). In addition, previous studies suggested that Fkh1 competes with a stable Fkh2/Mcm1 complex for occupancy at target promoters. In fact, Fkh1 seems to limit transcriptional activation of target genes, while Fkh2 plays an additional role in stabilizing Mcm1 at Fkh-controlled promoters. Furthermore, Fkh2 exploits a repressive role of *CLB2* transcription during early phases of the cell cycle (Koranda et al., 2000; Zhu et al., 2000) via interaction with another HDAC, the Sin3/Rpd3 complex (Hollenhorst et al., 2000; Ho et al., 2002; Veis et al., 2007; Voth et al., 2007), which it has been suggested to act as a boundary element for Sir2-dependent chromatin silencing at mating loci *HMR* and *HML* (Zhou et al., 2009). Deletion of *RPD3* leads to a decreased Sir2 level at telomeres and *HM* and to an increased Sir2 level at adjacent regions, resulting in Sir-dependent local propagation of transcriptional repression (Zhou et al., 2009). The Sin3/Rpd3 complex functions in a parallel pathway with the Isw2 chromatin remodeling ATPase (Fazzio et al., 2001). Interestingly, Isw2 and its homologue Isw1 repress *CLB2* expression via Fkh1 and Fkh2; Fkh1 acts with Isw1 to limit *CLB2* mRNA levels during the G2/M transition, whereas Fkh2 and Isw2 fully repress *CLB2* promoter during G1 phase (Sherriff et al., 2007). Altogether, these evidence indicate that Fkh transcription factors regulate both positively and negatively the Clb2 level critical for cell division, and suggest that Fkh-dependent recruitment of HDACs, and in particular of Sir2, could be a critical mechanism to fine tune G2/M gene expression required for cell division. Interestingly, Fkh1 could play a more prominent role in *CLB2* repression than Fkh2, due to the fact that, in exponentially growing cells, deletion of *FKH2* as well as the simultaneous disruption of *FKH2* and *SIR2* led to a decrease in *CLB2* mRNA levels as compared to *fkh1*Δ and *fkh1*Δ*sir2*Δ mutants. The possible role of Fkh1 and Fkh2 in these scenarios for the timing of Clb2 activation is currently under investigation.

The results presented in this work provide further evidence to support an evolutionary conserved role for the interplay between Fkh transcription factors and Sir2 in response to oxidative stress. Of note, a microarray analysis of oxidant-treated *FKH1* and *FKH2* deficient cells identified alterations in cell cycle and stress response genes (Zhu et al., 2000; Shapira et al., 2004). Mitotic active yeast cells exposed to H_2_O_2_were blocked in G2/M phase in an Fkh-dependent manner, and inactivation of Mcm1/Fkh2 was suggested to be responsible in mediating these effects (Shapira et al., 2004). In addition, deletion of both *FKH1* and *FKH2* has been shown to block normal lifespan and stress resistance of cells, especially in stationary phase, whereas overexpression of both genes extended stress resistance and chronological and replicative lifespan (Postnikoff et al., 2012). Strikingly, an additional role for Sir2 in lifespan determination upon caloric restriction (Guarente, 1999) as well as in oxidative stress resistance through activation of mitochondrial metabolism has been previously reported (Rodriguez-Colman et al., 2010; Sorolla et al., 2011). Here, we have shown that the interplay between Fkhs and Sir2 is stress responsive, with Fkh1 and Fkh2 involved in the protective role against oxidative stress mediated by Sir2, as shown for Hcm1 (Rodriguez-Colman et al., 2010). Sir2 was highly enriched at the *CLB2* promoter under starvation and stress conditions, and its binding as well as nuclear localization was dependent on Fkh1 and Fkh2. Moreover, *CLB2* transcript and protein levels were altered comparing *fkh1*Δ and *fkh2*Δ single deletion strains with *fkh1*Δ*fkh2*Δ, *fkh1*Δ*sir2*Δ, and *fkh2*Δ*sir2*Δ double deletion strains, suggesting that Fkh transcription factors act in concert with Sir2 to induce cell cycle arrest upon oxidative stress as well as at stationary phase. Our result that additional expression of Clb2 strongly affects the viability of yeast cells under oxidative stress conditions, and especially in stationary phase, indicates the importance of a tight control of Clb2 transcription. Thus, the Fkh/Sir2 association might suppress cell proliferation in response to oxidative stress in order to ensure the proper activation of pathways modulating stress response and longevity.

In agreement with our findings in yeast, also in mammalian cells SIRT1 associates with FOXO1, 3, and 4 under stress conditions (Brunet et al., 2004; Daitoku et al., 2004; Motta et al., 2004). This interplay promotes the nuclear import of these Fkh transcription factors and activation of genes involved in oxidative stress resistance (Brunet et al., 2004; van der Horst et al., 2004). Mammalian FoxO3 is similar to yeast Hcm1 and is involved in cell cycle, aging and cancer (Murakami et al., 2010). Furthermore, the SIRT6/7 homolog SIR-2.4 in *C. elegans* promotes relocalization and function of the FOXO transcription factor DAF-16 during stress (Chiang et al., 2012). In this regard, the functional human homolog of Fkh1 and Fkh2, FoxM1, plays a role in maintaining cell survival under oxidative and heat stress conditions (Li et al., 2008; Park et al., 2009) and regulates transcription of cyclin B1, the human homolog of yeast Clb2 (Murakami et al., 2010; Laoukili et al., 2005).

Furthermore, our finding that Sir2 overexpression affected cell growth in the presence of both Fkh1 and Fkh2 highlights their potential role in suppressing genes controlling cell cycle progression. Here we hypothesize that a temporal reduction in the number of cell divisions of a single mitotic cell for a definite environmental stress period could lead to an extension of its chronological and replicative lifespan. In this light, the extended chronological lifespan of yeast cells observed upon increased Sir2/Fkh activity is probably facilitated by a reduced cell cycle progression in their replicative stage as well. In support to this, it has been reported that Sir2 levels rise in response to oxidative stress (Sorolla et al., 2011) and that its increased activity might have a protective role through activation of genes involved in stress resistance (Fabrizio et al., 2005; Rodriguez-Colman et al., 2010). Thus, Sir2-mediated chromatin silencing via Fkh1 and Fkh2 provides an additional level of regulation of the cell division cycle, which is also required for an increased stress resistance, as previously suggested for Fkhs (Shapira et al., 2004; Rodriguez-Colman et al., 2010; Postnikoff et al., 2012). Moreover, Fkh transcription factors might help to recruit Sir2 and other HDACs to maintain genome stability, consistently with previous studies demonstrating that Sir2 silences heterochromatin structures like telomeres, nucleolar rDNA, and mating type loci (Bitterman et al., 2002).

### Conflict of interest statement

The authors declare that the research was conducted in the absence of any commercial or financial relationships that could be construed as a potential conflict of interest.

## Research Topic:

From structural to molecular systems biology: experimental and computational approaches to unravel mechanisms of kinase activity regulation in cancer and neurodegeneration

## Topic Editor:

Matteo Barberis, Humboldt University Berlin, Germany; Max Planck Institute for Molecular Genetics, Berlin, Germany
